# Unveiling protein corona composition: predicting with resampling embedding and machine learning

**DOI:** 10.1093/rb/rbad082

**Published:** 2023-12-12

**Authors:** Rong Liao, Yan Zhuang, Xiangfeng Li, Ke Chen, Xingming Wang, Cong Feng, Guangfu Yin, Xiangdong Zhu, Jiangli Lin, Xingdong Zhang

**Affiliations:** College of Biomedical Engineering, National Engineering Research Centre for Biomaterials, Sichuan University, Chengdu, 610065, China; College of Biomedical Engineering, National Engineering Research Centre for Biomaterials, Sichuan University, Chengdu, 610065, China; College of Biomedical Engineering, National Engineering Research Centre for Biomaterials, Sichuan University, Chengdu, 610065, China; College of Biomedical Engineering, National Engineering Research Centre for Biomaterials, Sichuan University, Chengdu, 610065, China; College of Biomedical Engineering, National Engineering Research Centre for Biomaterials, Sichuan University, Chengdu, 610065, China; College of Biomedical Engineering, National Engineering Research Centre for Biomaterials, Sichuan University, Chengdu, 610065, China; College of Biomedical Engineering, National Engineering Research Centre for Biomaterials, Sichuan University, Chengdu, 610065, China; College of Biomedical Engineering, National Engineering Research Centre for Biomaterials, Sichuan University, Chengdu, 610065, China; College of Biomedical Engineering, National Engineering Research Centre for Biomaterials, Sichuan University, Chengdu, 610065, China; College of Biomedical Engineering, National Engineering Research Centre for Biomaterials, Sichuan University, Chengdu, 610065, China

**Keywords:** nanoparticles, protein corona, machine learning, resampling technique, feature analysis

## Abstract

Biomaterials with surface nanostructures effectively enhance protein secretion and stimulate tissue regeneration. When nanoparticles (NPs) enter the living system, they quickly interact with proteins in the body fluid, forming the protein corona (PC). The accurate prediction of the PC composition is critical for analyzing the osteoinductivity of biomaterials and guiding the reverse design of NPs. However, achieving accurate predictions remains a significant challenge. Although several machine learning (ML) models like Random Forest (RF) have been used for PC prediction, they often fail to consider the extreme values in the abundance region of PC absorption and struggle to improve accuracy due to the imbalanced data distribution. In this study, resampling embedding was introduced to resolve the issue of imbalanced distribution in PC data. Various ML models were evaluated, and RF model was finally used for prediction, and good correlation coefficient (*R*^2^) and root-mean-square deviation (RMSE) values were obtained. Our ablation experiments demonstrated that the proposed method achieved an *R*^2^ of 0.68, indicating an improvement of approximately 10%, and an RMSE of 0.90, representing a reduction of approximately 10%. Furthermore, through the verification of label-free quantification of four NPs: hydroxyapatite (HA), titanium dioxide (TiO_2_), silicon dioxide (SiO_2_) and silver (Ag), and we achieved a prediction performance with an *R*^2^ value >0.70 using Random Oversampling. Additionally, the feature analysis revealed that the composition of the PC is most significantly influenced by the incubation plasma concentration, PDI and surface modification.

## Graphical Abstract

**Figure:**
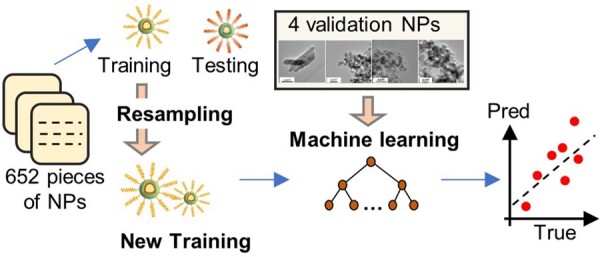

## Introduction

After the biomaterial is implanted in the body, some specific proteins (such as fibronectin, vitronectin and laminin) are adsorbed on the surface of the implant before the cells attach to it, which further regulates the behavior of the cells and affects tissue regeneration [1]. The presence of surface nanostructures on biomaterials profoundly alters their interaction with proteins. Studies have shown that biomaterials with surface nanostructures can promote greater protein secretion and stimulate new bone growth more effectively than conventional ones [2, 3]. After the nanoparticles (NPs) enter the living system, proteins in body fluids (such as blood) are rapidly adsorbed to the surface of the NPs, forming the protein corona (PC) [4]. It is the PC, rather than the size, shape and surface chemistry of the NPs, that determines their physical and chemical identity. Analyzing the impact of NP’s physical and chemical properties on PC composition is crucial for rational utilization of the PC and ultimately improving the physiological functions of NPs. Significant progress has been made in the research of the PC, utilizing various analysis methods [5, 6]. Mature protein structure analysis techniques include dynamic light scattering (DLS), differential centrifugal sedimentation [7, 8] and transmission electron microscopy (TEM) [9]. The relative protein abundance (RPA) can be quantitatively assessed using methods like bicinchoninic acid assay and Bradford method of determination [10, 11]. Conformational changes in the PC can be detected through Fourier transform infrared spectroscopy and so on [12, 13]. Duan *et al.* [14] introduced fluorescamine to label fluorescence change as a novel descriptor for engineering nanoparticles (ENPs). They combined it with conventional descriptors of protein (such as isoelectric point and hydrophobicity value) and then used Random Forest (RF) regression model to predict changes in the PRA. Findlay MR applied ENPs properties, proteins characteristics and solution conditions for PC formation to classify whether ENPs are associated with proteins, achieving an F1-score of 0.81 [15]. Helma *et al.* [16] employed a PC dataset as descriptors to predict the toxicities of NPs, achieving an *R*^2^ of 0.68. Ban *et al.* [17] utilized the RF model to predict the RPA of individual proteins, achieving good *R*^2^. However, the researches above mainly focus on the prediction model, less on the imbalanced distribution of data itself. We notice that the quality of data actually affects the model performance to a great extent. Resampling technology can be used to change the distribution of dense and sparse samples. This article applies this technology to predict PC to solve the problem of imbalanced data distribution with high accuracy.

## Materials and methods

In this section, we will provide a detailed description of our data source and the resampling methods employed. Subsequently, we will present our machine learning (ML) models for predicting the RPA of individual proteins. Next, we will introduce the evaluation methods utilized to assess the performance of our model. Finally, we will conduct validation experiments involving four distinct NPs to validate the efficacy of our method.

### Data

**Figure 1.**
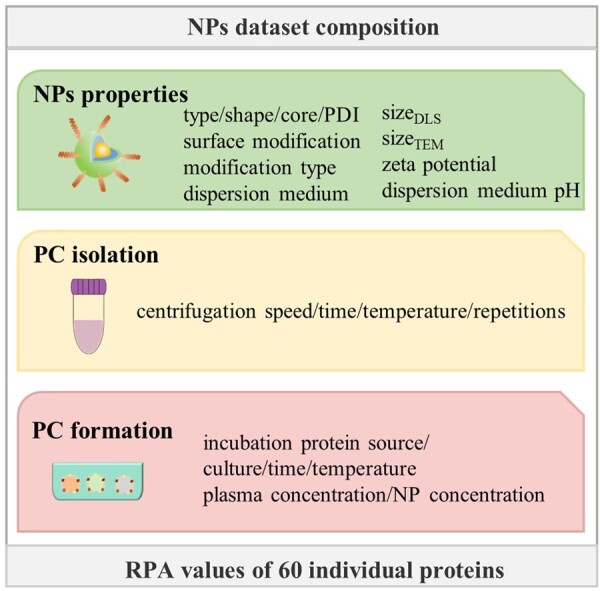
Twenty-one factors of the datasets. The features of NPs dataset consist of three parts: the properties of NPs, isolation of PC and formation of PC.

A total of 652 data were obtained from Ban Z’s open data [17], where each record having 21 factors as model input parameters, and was complete without any missing data. In our study, we specifically focused on the RPA values of 60 proteins within this dataset. The 21 factors, as illustrated in Figure 1, including NP properties (NP type, NP core, surface modification, modification type, size measured by DLS (size_TEM_), size measured by DLS (size_DLS_), zeta potential and polydispersity index (PDI, NP shape, dispersion medium, dispersion medium pH). Additionally, it encompassed factors related to PC isolation, such as centrifugation speed, centrifugation time, centrifugation temperature and centrifugation repetitions. Furthermore, factors associated with PC formation are included, such as protein source, incubation culture, incubation plasma concentration, incubation NP concentration, incubation time and incubation temperature.

We used two approaches to process the data of the 21 factors. The eight category factors were encoded by OneHot-Encoding. The remaining 13 numerical factors were transferred by Max-Min Normalization maintaining the proportion relationship of the original data to a certain extent, as flows form the formula:

(1)  X*=Xi-XminXmax-Xmin

where $X^{*}$ is the normalized value and $X_{i}$ is the original data. $X_{\max}$ is the maximum value and $X_{\min}$ is the minimum value in the original data.

To ensure almost identical distributions between the train set and test set, we employ stratified sampling to divide the data into the 9:1 ratio, creating training and testing datasets.

### Resampling methods

To address the issue of data imbalance, we applied resampling techniques, which involves using certain algorithms to generate new samples from the existing data. In this article, three resampling methods were applied: Random Oversampling, Synthetic Minority Oversampling Technique for Regression (SmoteR) and Weighted Relevance-based Combination Strategy (WERCS) [18–20].

The relevance function serves as the foundation for data resampling. The relevance function [19] φ(Y):Y → [0,1] is a continuous function that expresses the application-specific bias concerning the target variable domain Y by mapping it into a [0, 1] scale of relevance, where 0 and 1 represent the minimum and maximum relevance, respectively. Using the relevance function can distinguish between sparse and dense “classes”. For imbalanced data, given the potentially infinite nature of the target variable domain, it is impractical to calculate all values, requiring an approximation. Two essential parts are necessary:

1. A set of data points where relevance is known (control points). The set ${S=\{<y_{k},\varphi (y_{k}),\varphi^{\prime }(y_{k})>\}}_{k=1}^{S}$ must be given as input to an interpolation algorithm. The element in the set S is (i) the target value $y_{k}$, (ii) its relevance value $\varphi \left(y_{k}\right)$ and (iii) the first derivative of the relevance function at the point $\varphi^{\prime }\left(y_{k}\right)$. By default, control points are assumed as local minimum or maximum of the relevance, thus the derivative values are equal to zero.
2. A decision on which interpolation method to use. As in early work by Ribeiro, the use of Piecewise Cubic Hermite Interpolating Polynomials [21] (*pchip*) to approximately gain the relevance function. Table 1 shows how *pchip* does on a set of control points S [19].

**Table 1.** Piecewise cubic hermite interpolating polynomial

Algorithm 1 *pchip*(S) Piecewise Cubic Hermite Interpolating Polynomial.
**Input:** ${S=\{〈y_{k},\varphi (y_{k}),\varphi^{\prime }(y_{k})〉\}}_{k=1}^{S}$ ,set of control points with $y_{1}$ < $y_{2}<...<y_{s}$, their relevance values $\varphi \left(y_{k}\right)$, and first derivative $\varphi^{\prime }\left(y_{k}\right)$. **Output:** Φ(y): Piecewise Cubic Hermite Interpolating Polynomial.
1: **for** k ← 1 **to** s-1 **do** 2: $h_{k}\leftarrow y_{k+1}-{\;y}_{k}$ 3: $\delta_{k}\leftarrow \left(\varphi (y_{k+1})-\varphi (y_{k})\right)/h_{k}$ 4: $a_{k}\leftarrow \varphi (y_{k}$) 5: **end for** 6: ${\left\{b_{k}\right\}}_{k=1}^{s-1}\leftarrow$check_slopes (${\left\{\varphi^{\prime }\left(y_{k}\right)\right\}}_{k=1}^{s-1}$), ${\left\{\delta_{k}\right\}}_{k=1}^{s-1}$) check_slopes [22] 7: **for** k ← 1 **to** s-1 **do** 8: $c_{k}$$\leftarrow \;\left({3\delta }_{k}-{2b}_{k}+b_{k+1}\right)/h_{k}$ 9: $d_{k}$$\leftarrow \;\left(b_{k}-{2\delta }_{k}+b_{k+1}\right)/h_{k}^{2}$ 10: **end for** 11: **return**$\Phi (y)=a_{k}+b_{k}(y-y_{k})+c_{k}{\left(y-y_{k}\right)}^{2}+d_{k}{\left(y-y_{k}\right)}^{3},\;y\in [y_{k},y_{k+1}]$

The Random Oversampling method randomly selects some samples from the original data and adds them to train. These replicas are only introduced in the most important ranges of the target variable, i.e. in the ranges where the relevance Φ(y) is a threshold. The aim is to better balance the number of the majority and minority samples.

The SmoteR is a random method combining *K* nearest neighbors. The basic process is to randomly select a sample X in a minority class ($D_{r}$) and calculate *K* nearest neighbors of the samples; and then stochastically select a sample O from *K* nearest neighbors, calculating the product of the random number between it and (0,1) as a new sample without repetition. This algorithm will oversample the observations in $D_{r}$, thus leading to a new train set with a more balanced distribution of the values. Supplementary Tables S1 and S2 show the main details of SmoteR [23, 24].

The WERCS method uses the correlation function as the probability of resampling. The high correlation data will likely be added to the original samples as new data. In oversampling, examples with higher relevance have a higher probability of being replicated. In Undersampling, examples are randomly selected to be removed with probability 1−Φ(y), the higher the relevance value of an example, the lower will be the probability of being removed. The parameters of three resampling are in Supplementary Table S3.

#### Random Forest model

**Figure 2.**
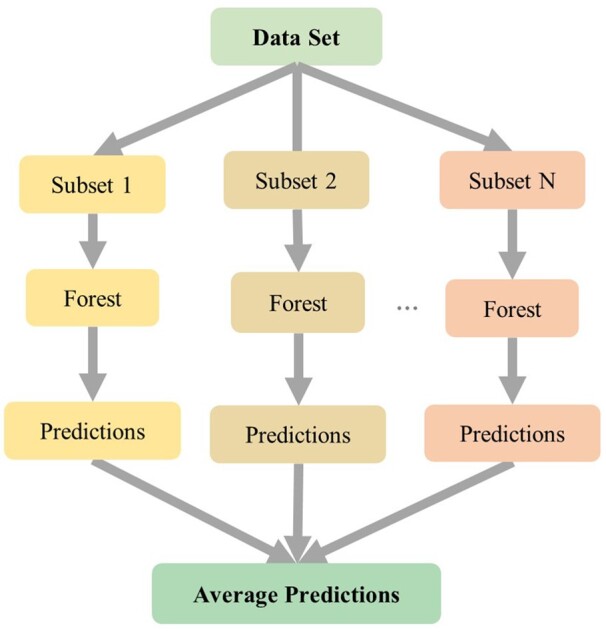
The prediction flow of RF model. The prediction of the RF model was the average of the regression results for all trees.

We tried three nonlinear ML models: SVR, RF and MLP models, and RF to gain better performance with the lowest root-mean-square deviation (RMSE). The details of the models are in Supplementary Tables S4–S6. RF is first proposed in 2001 and can be used to solve classification and regression problems. It is an integrated learning model. For regression problems, the final prediction results come from the mean of the predicted values of all trees in the model. The idea of RF model is to combine bagging strategy and decision trees. Bagging is derived from the bootstrap method, which uses bootstrap to sample the train set samples and then combines them with the decision tree to decide the final output through the voting of the decision tree shown in Figure 2.

#### Evaluation metrics

To evaluate the regression prediction performance of various ML models, three evaluation metrics are employed: *R*^2^, RMSE and learning curves. In the subsequent calculation formulas (2) and (3), the unified parameters are defined as follows: the model predictive value is denoted as *p*, the true value as *r* and *n* represents the number of samples. Specifically, $r_{i}$ represents the true value of the *i*-th sample, $p_{i}$ represents the predictive value of *i*-th sample, $\bar{r}$ signifies the average of the true value across all *n* samples and $\bar{p}$ represents the average of the predictive values of all *n* samples.

(2)  R2=∑i=0n(ri-r¯)(pi-p¯)∑i=0n(ri-r¯)2∑i=0npi-p¯2  

(3)  RMSE=1n∑i=0n(ri-pi)2

Learning curves can be used to determine whether the model is overfitting or underfitting the current data. These curves plot the score changes as the number of cross-validation data increases. For a well-trained model, the cross-validation score shows a certain trend improvement with the increase of data.

Variance and entropy are metrics used to characterize data distribution. Variance quantifies the extent of data dispersion, with higher values indicating greater dispersion and a lack of significant data concentration. On the other hand, entropy serves measure as a measure of uncertainty or confusion within variables. A higher entropy value suggests a more uniform distribution of data.

### Validation experiment

We prepared four NPs including the most common metal oxide NP classes: SiO_2_, TiO_2_ and most common metal class of NP: Ag, and the non-metal class of NP: hydroxyapatite (HA), which played an important role in stimulating tissue regeneration to validate our model. And HA NP was not in the original dataset so as to verify the generalization ability of our model for the new sample.

Our testing method for the adsorbed proteins was label-free quantification (supported by Allwegene Tech.). This technology emerged as an important mass spectrometry (MS) quantitative method [25, 26], which analyzed changes in the amount of protein in samples from different sources by comparing the intensity of MS peaks. The frequency with which peptides are captured and detected by MS is positively correlated with their abundance in a mixture, so the number of counts of proteins detected by MS reflects their abundance, and the proteins are quantified.

The experiment conditions were as follows: the HA sample was supplied by Sichuan Baiamung Bioactive Materials Co., Ltd. And TiO_2_, SiO_2_ and Ag samples were supplied by Nanjing XFNANO Materials Tech Co., Ltd. The Kel was supplied by College of Biomedical Engineering of Sichuan University. Kel serum was thawed from −20 to 4°C as a backup the day before the experiment. A 10 mg of each kind of NPs were incubated with 10 ml of Kel serum at 37°C for 2 h and then centrifuged at room temperature with a 15 970×*g* centrifuge (Shuke TG-18) at high speed to remove the supernatant, resuspended with water, centrifuged, washed again, centrifuged twice in total and finally stored at −80°C after being extremely cooled by liquid nitrogen for 15 min, finally used as the sample for Label-free analysis for standby. The material was characterized using transmission electron microscopy (HRTEM was conducted on a Hitachi H-800 operating at 200 kV), and the zeta potential was measured using Malvern instruments.

## Results and discussion

In this section, we will present the results of resampling on training data, the performance of the RF model, the outcomes of four NPs experiments involving model prediction, and the feature importance analysis of the models.

### Training data resampling results

**Figure 3.**
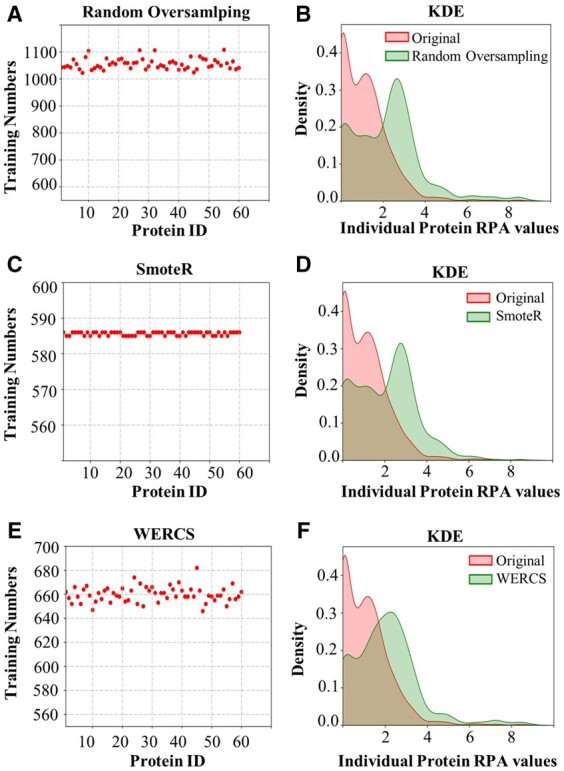
The left column (**A**) (**C**) (**E**) shows the number of training set for 60 individual proteins resampled by Random Oversampling, SmoteR and WERCS, respectively. The right column (**B**) (**D**) (**F**) shows the KDE of the 1st protein in all data of the original and resampled data by Random Oversampling, SmoteR, and WERCS, respectively.

Detailed parameters of three resampling methods are shown in Supplementary Table S3. Here, Figure 3 shows the results of the three resampling techniques applied to the training data. As depicted in Figure 3, the distribution of data becomes more balanced after the three resampling methods. In Figure 3A, C and E, the *x*-axis represents 60 individual proteins, while the *y*-axis represents the sample size of each protein after resampling. Figure 3B, D and F displays the Kernel Density Estimation (KDE) of 60 individual proteins’ training set after undergoing the three resampling methods, with the red line representing the resampled data and the green line representing the original training data. As shown in Figure 3, the original distribution of RPA values is skewed with positive skewness and a long tail extending to the right. After implementing the three resampling methods, the distribution of this data has been improved, leading to an increase of data points with low density and a decrease in data points with high density in some way, achieving a more balanced effect than the original training data.

Two statistical indicators were used to compare the distribution of PC before and after resampling. The variance of the resampled PC increased, suggesting a more dispersed and less concentrated distribution of the PC data. Additionally, the entropy indicator increased, and more evenly distributed PC data. These findings are illustrated in Table 2.

**Table 2.** The mean values of the variance and entropy indicators before and after resampling on 60 individual proteins

Original + resampled	Variance	Entropy
Original	2.71	5.06
Original + Random Oversampling	**5.62**	**6.23**
Original + SmoteR	**4.91**	**5.59**
Original + WERCS	**5.84**	**5.80**

The bolded values in this table indicate that our method is improving and effective on the original metrics.

### Model predicting results

**Figure 4.**
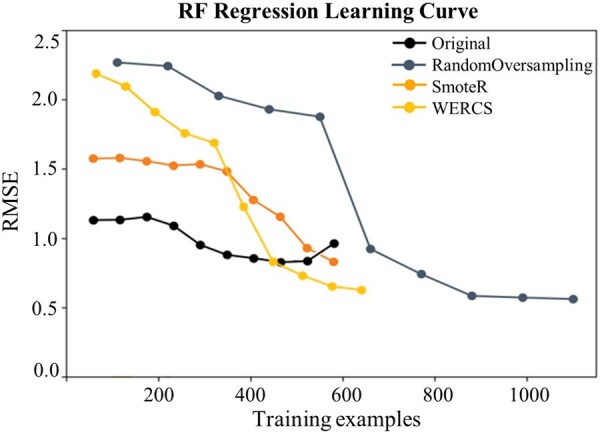
The RMSE Index curves of RF model from the original and resampled data by Random Oversampling, SmoteR and WERCS on cross-validation data, respectively.

In this section, we showed the evaluation results of the RF model after resampling. We used the results of RF model without resampling as the baseline. Figure 4 shows the 10-fold cross-validation learning curves of RF model on the training data of 60 individual proteins. It could be seen that the baseline RF model with resampling achieving better performance, especially Random Oversampling resampling, and the RMSE indicator on the cross-validation set basically decreased with the increase of train samples >600, which indicated the model trending to be stable.

**Figure 5.**
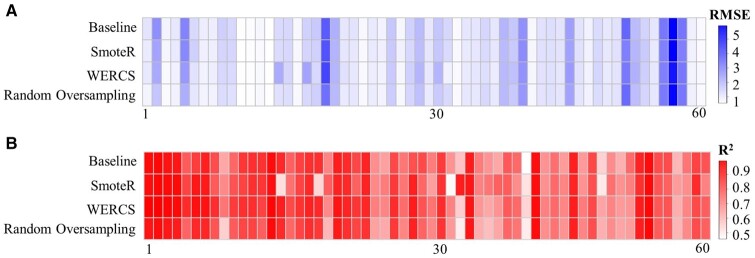
The Comparison of RMSE and *R*^2^ before and after resampling. (**A**) is the RMSE of each protein and (**B**) is the *R*^2^ of each protein.

As shown in Figure 4, the RMSE on the model with Random Oversampling, SmoteR and WERCS had been decreased due to the weight of training data after resampling, which was the possible reason why the model had a better performance. And Table 3 shows the mean values of RMSE and *R*^2^ of RF model before and after three resampling for 60 individual proteins, and Figure 5 shows the results of each protein which could also show the improvement of using resampling method with ∼10% improvement on *R*^2^ and 10% reduction on RMSE.

**Table 3.** The mean values of RMSE and *R*^2^ of RF model before and after three resampling for 60 individual proteins

Model + resampling	*R* ^2^	RMSE
RF (baseline)	0.62	1.01
RF + SmoteR	**0.65**	**0.97**
RF + WERCS	**0.64**	**0.99**
RF + Random Oversampling	**0.68 (+0.06)**	**0.90 (−0.11)**

The bolded values in this table indicate that our method is improving and effective on the original metrics.

### Feature importance analysis

In order to further know the important features, we use RF model to rank the importance of features. RF model calculates the importance of features through the Gini index to score the importance of 21 influencing factors after pretreatment. We filter out features with importance >0.01 of 21 features on 60 target individual proteins. Then, the selected characters of the 21 features are classified. Finally, the most important factors affecting 60 proteins RPAs are shown in Table 4. The most important features are incubation plasma concentration, PDI and surface modification, which importance are 0.27, 0.26 and 0.23, respectively.

**Table 4.** Feature importance of 60 individual proteins

Feature	Importance
Incubation plasma concentration	**0.27**
PDI	**0.26**
Surface modification	**0.23**
NP without modification	0.22

The bolded values in this table indicate that our method is improving and effective on the original metrics.

## Four NPs experiments

**Figure 6.**
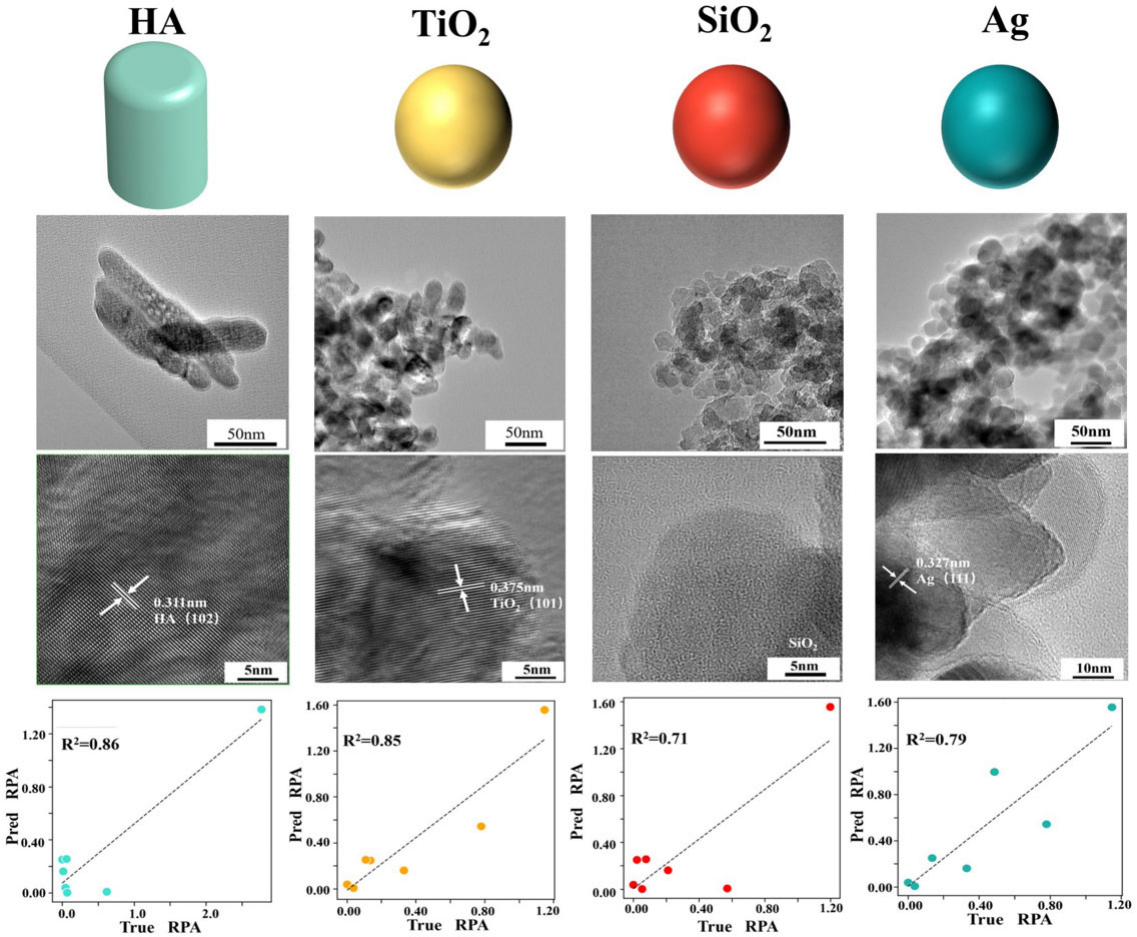
The Performance on *R*^2^ of four extra NPs experiments. The diagrams in the first row represented the four particle schematics of our experiments, and the diagrams in the second and third rows showed the high-magnification and low-magnification TEM images of these NPs. And the grams in the last row showed the model performance of *R*^2^. HA, TiO_2_ and Ag exhibited significant crystal structures with obvious streaks, while the SiO_2_ exhibited a significant amorphous structure.

As described in Validation experiment section, we selected the seven individual proteins with high abundance as the model predictive targets. As shown in Figure 6, the validation results showed that our model achieved a good prediction effect with *R*^2^ >0.70.

However, for those proteins with low RPA values of individual proteins, our model performance is not good, which may be caused by the competitive adsorption between different proteins in our proteomics experiment. In our experiment, we did not analyze many proteins and chose the seven representative proteins (ID: P01834, P08603, P02655, Q14520, P01008, P01857 and P0C0L4) which were associated with the immune response [27] and possibly affect tissue regeneration. In terms of these proteins as extra verified samples, our model could predict their RPAs accurately with the *R*^2^ >0.70. In a word, the prediction score gained in the experiment of NPs largely resulted from the fact that we reached a better training performance using the resampling method. Ablation experiments on resampling could increase the *R*^2^ by 0.06 and reduce the RMSE by 0.11 to the greatest extent possible, which again illustrated the importance of data processing.

Moreover, in order to achieve better prediction results, we should start from the following points in the future: increase the collection of the data based on this; second, with enough data, we can consider using the deep learning model of tabular data [28–30], which may have higher accuracy under a large amount of data.

The formation of PC over a long period of time could affect the biological responses of nanomaterials, such as biosorption and biotoxicity [31]. PC might also attenuate the primitive cytotoxicity of NPs [32, 33]. This was mainly due to the protective effect of PC against NP-induced cellular damage. The interaction of naked NPs with the cell disrupted the integrity of the plasma membrane and led to cell rupture, whereas the protein coverage made the surface of NPs more biocompatible and reduced the damage of NPs to the cell membrane [34–36]. Therefore, our work would consider collecting new data from published literature on the NPs and biological effects to further validate the effectiveness of our resampling method in the future.

## Conclusion

The resampling method has significantly improved the prediction error of the RMSE indicator by 10% points, suggesting the data resampling has a substantial positive impact on the prediction model. This means the quality of data greatly affecting the performance of the model. Additionally, the feature analysis showed that incubation plasma concentration, PDI and surface modification are the three most influential features affecting the RPA values for individual proteins calculated by the prediction model. Our model, trained using resampled data, demonstrated a good performance in predicting PC compositions, a validation further supported by the label-free proteomics experiment on four NPs: TiO_2_, SiO_2_, HA and Ag. The prediction results for the RPA of proteins using these four NPs showed an *R*^2^ value >0.70.

## Supplementary Material

rbad082_Supplementary_Data
