# Supplementary material for: Unveiling protein corona composition: predicting with resampling embedding and machine learning
Source: Regen Biomater. 2023 Dec 12;11:rbad082. doi: 10.1093/rb/rbad082 (PMC10781662; doi:10.1093/rb/rbad082)

Size Distribution Report by Volume

v2.2

Sample Details

Sample Name: 4 1

SOP Name: WYY.sop

General Notes:

**File Name:**	20230302.dts	**Dispersant Name:**	Water
**Record Number:**	5	**Dispersant RI:**	1.330
**Material RI:**	1.59	**Viscosity (cP):**	0.8872
**Material Absorbtion:**	0.010	**Measurement Date and Time:**	2023年3月2日 21:10:39

System

**Temperature (°C):**	25.0	**Duration Used (s):**	60
**Count Rate (kcps):**	320.5	**Measurement Position (mm):**	4.65
**Cell Description:**	Disposable sizing cuvette	**Attenuator:**	9

Results

			Size (d.nm):	% Volume:	St Dev (d.nm):
**Z-Average (d.nm):**	147.6	**Peak 1:**	131.4	8.4	61.98
**PdI:**	0.351	**Peak 2:**	20.00	85.6	4.520
**Intercept:**	0.798	**Peak 3:**	5441	6.0	640.9

Result quality **Refer to quality report**

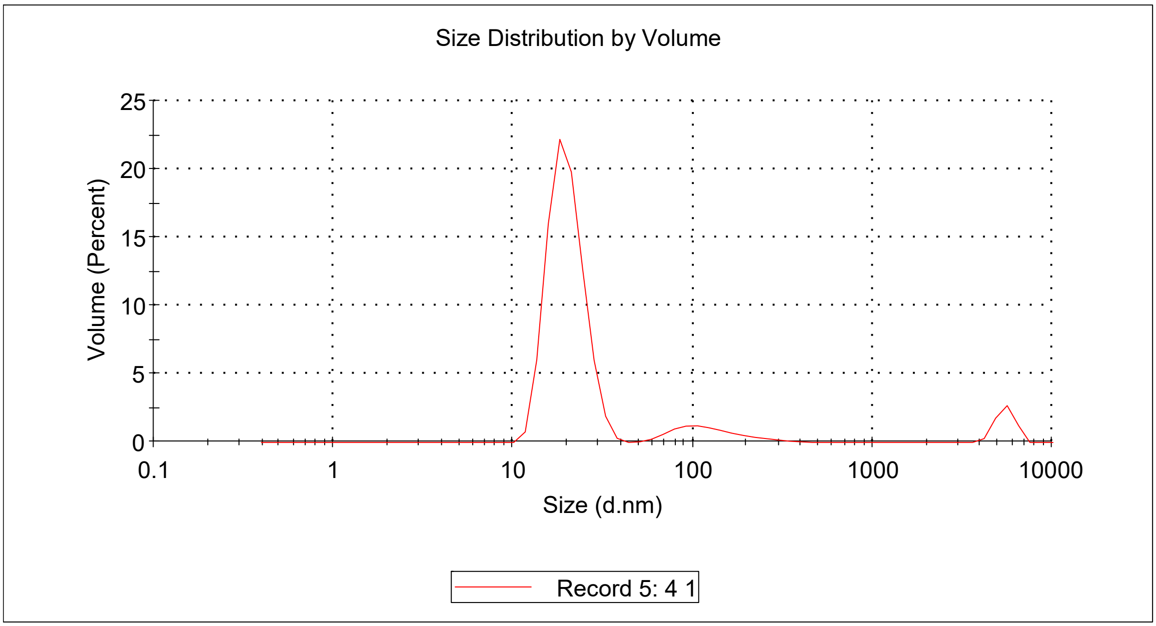

Supplement: rbad082_Supplementary_Data [file rbad082_supplementary_data.zip › DLS-Ag.pdf]
