# Supplementary material for: Unveiling protein corona composition: predicting with resampling embedding and machine learning
Source: Regen Biomater. 2023 Dec 12;11:rbad082. doi: 10.1093/rb/rbad082 (PMC10781662; doi:10.1093/rb/rbad082)

# Size Distribution Report by Volume

*v2.2*

## Sample Details

**Sample Name:** 1 1

**SOP Name:** WYY.sop

**General Notes:**

| | | | |
|---|---|---|---|
| **File Name:** | 20230302.dts | **Dispersant Name:** | Water |
| **Record Number:** | 1 | **Dispersant RI:** | 1.330 |
| **Material RI:** | 1.59 | **Viscosity (cP):** | 0.8872 |
| **Material Absorbtion:** | 0.010 | **Measurement Date and Time:** | 2023年3月2日 20:49:35 |

## System

| | | | |
|---|---|---|---|
| **Temperature (°C):** | 25.0 | **Duration Used (s):** | 60 |
| **Count Rate (kcps):** | 268.6 | **Measurement Position (mm):** | 4.65 |
| **Cell Description:** | Disposable sizing cuvette | **Attenuator:** | 9 |

## Results

| | | | Size (d.nm): | % Volume: | St Dev (d.nm): |
|---|---|---|---|---|---|
| **Z-Average (d.nm):** | 1946 | **Peak 1:** | 2439 | 100.0 | 735.2 |
| **PdI:** | 0.273 | **Peak 2:** | 0.000 | 0.0 | 0.000 |
| **Intercept:** | 0.942 | **Peak 3:** | 0.000 | 0.0 | 0.000 |
| **Result quality** | **Good** | | | | |

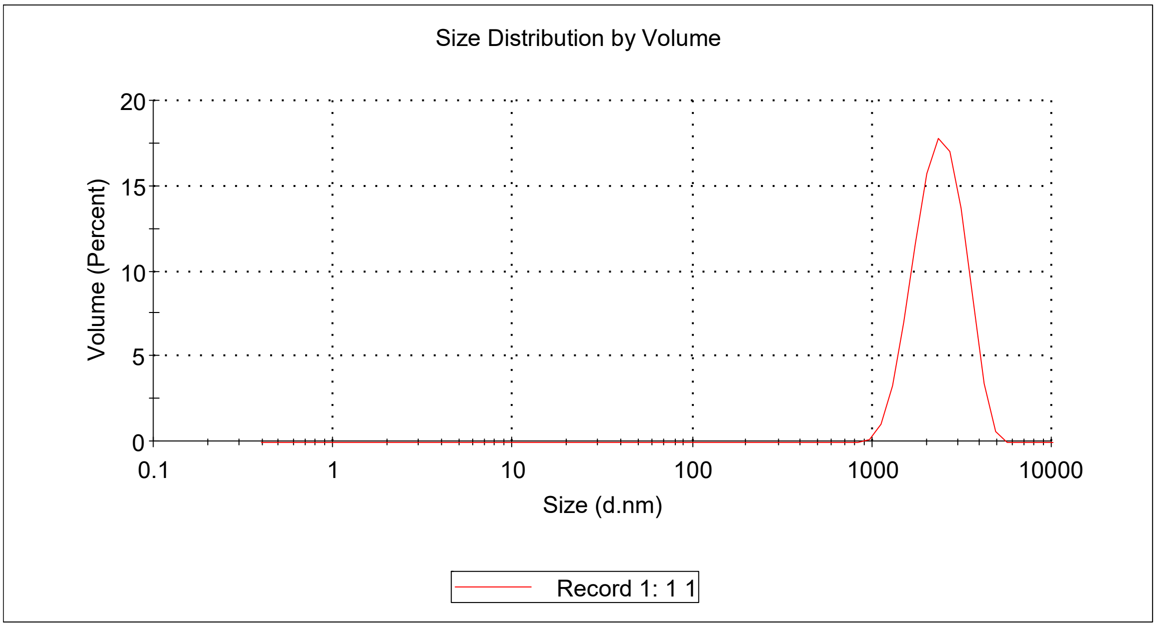

Supplement: rbad082_Supplementary_Data [file rbad082_supplementary_data.zip › DLS-HA.pdf]
