# Supplementary material for: Unveiling protein corona composition: predicting with resampling embedding and machine learning
Source: Regen Biomater. 2023 Dec 12;11:rbad082. doi: 10.1093/rb/rbad082 (PMC10781662; doi:10.1093/rb/rbad082)

Size Distribution Report by Volume

v2.2

Malvern

Sample Details

Sample Name: 3 1

SOP Name: WYY.sop

General Notes:

**File Name:**	20230302.dts	**Dispersant Name:**	Water
**Record Number:**	4	**Dispersant RI:**	1.330
**Material RI:**	1.59	**Viscosity (cP):**	0.8872
**Material Absorbtion:**	0.010	**Measurement Date and Time:**	2023年3月2日 21:05:25

System

**Temperature (°C):**	25.0	**Duration Used (s):**	60
**Count Rate (kcps):**	288.5	**Measurement Position (mm):**	4.65
**Cell Description:**	Disposable sizing cuvette	**Attenuator:**	10

Results

			**Size (d.nm):**	**% Volume:**	**St Dev (d.nm):**
**Z-Average (d.nm):**	535.7	**Peak 1:**	380.8	12.4	100.4
**PdI:**	0.420	**Peak 2:**	3825	87.6	1456
**Intercept:**	0.962	**Peak 3:**	0.000	0.0	0.000
**Result quality**	**Good**				

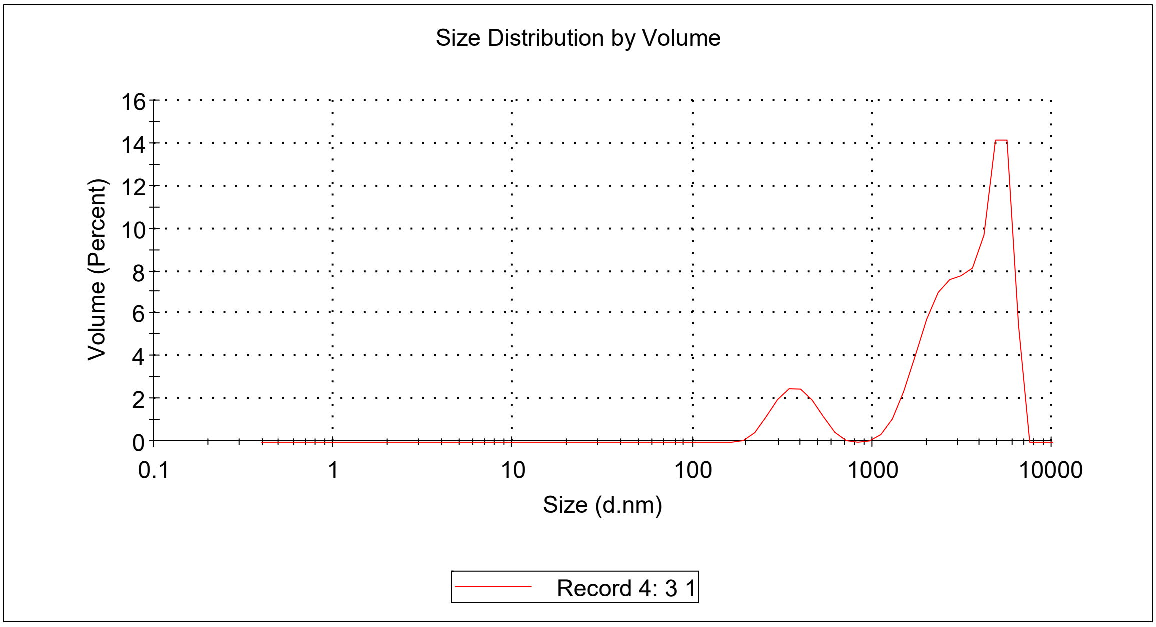

Supplement: rbad082_Supplementary_Data [file rbad082_supplementary_data.zip › DLS-SiO2.pdf]
